# Investigating and managing neonatal seizures in the UK: an explanatory sequential mixed methods approach

**DOI:** 10.1186/s12887-020-1918-4

**Published:** 2020-01-28

**Authors:** Lucy Gossling, James J. P. Alix, Theocharis Stavroulakis, Anthony R. Hart

**Affiliations:** 1 University of Sheffield Medical School, Beech Hill Road, Sheffield, S10 2RX UK; 2 Department of Neuroscience, University of Sheffield, Sheffield Institute for Translational Neuroscience, 385a Glossop Road, Sheffield, S10 2HQ UK; 3 Department of Paediatric and Neonatal Neurology, Sheffield Children’s Hospital NHS Foundation Trust, Ryegate Children’s Centre, Tapton Crescent Road, Sheffield, S10 5DD UK

**Keywords:** Infant / newborn, Seizures, Anticonvulsants, Differential diagnosis, Neurophysiology, Hypoxia-ischemia, Brain

## Abstract

**Background:**

Neonatal seizures are difficult to diagnose and, when they are, tradition dictates first line treatment is phenobarbital. There is little data on how consultants diagnose neonatal seizures, choose when to treat or how they choose aetiological investigations or drug treatments. The purpose of this study was to assess the variation across the UK in the management of neonatal seizures and explore paediatricians’ views on their diagnosis and treatment.

**Methods:**

An explanatory sequential mixed methods approach was used (QUAN→QUAL) with equal waiting between stages. We collected quantitative data from neonatology staff and paediatric neurologists using a questionnaire sent to neonatal units and via emails from the British Paediatric Neurology Association. We asked for copies of neonatal unit guidelines on the management of seizures. The data from questionnaires was used to identify16 consultants using semi-structured interviews. Thematic analysis was used to interpret qualitative data, which was triangulated with quantitative questionnaire data.

**Results:**

One hundred questionnaires were returned: 47.7% thought levetiracetam was as, or equally, effective as phenobarbital; 9.2% thought it was less effective. 79.6% of clinicians had seen no side effects in neonates with levetiracetam. 97.8% of unit guidelines recommended phenobarbital first line, with wide variation in subsequent drug choice, aetiological investigations, and advice on when to start treatment. Thematic analysis revealed three themes: *‘Managing uncertainty with neonatal seizures’*, *‘Moving practice forward’* and *‘Multidisciplinary team working’*. Consultants noted collecting evidence on anti-convulsant drugs in neonates is problematic, and recommended a number of solutions, including collaboration to reach consensus guidelines, to reduce diagnostic and management uncertainty.

**Conclusions:**

There is wide variation in the management of neonatal seizures and clinicians face many uncertainties. Our data has helped reveal some of the reasons for current practice and decision making. Suggestions to improve certainty include: educational initiatives to improve the ability of neonatal staff to describe suspicious events, greater use of video, closer working between neonatologists and neurologists, further research, and a national discussion to reach a consensus on a standardised approach to managing neonatal epileptic seizures.

## Background

Seizures are common in the neonatal period because of the relative excitability of the neonatal brain and high risk of pathologies leading to acute symptomatic seizures [1–7]. The true incidence of neonatal seizures is unknown, but proposed rates are 3/1000 term live births and between 132/1000 preterm neonates [8] One reason why incidence figures may be inaccurate is because neonatal seizures are difficult to diagnose: multiple studies using EEG have shown that most neonatal seizures have no clinical features at all (electrographic seizures) [9–14], and the accurate differentiation of epileptic seizures from non-epileptic events based on clinical skills alone is poor [9, 10, 15, 16]. For example, one study of term neonates showed only 34% of neonatal seizures having had clinical features and 73% of suspected seizures having had no epileptiform discharges associated with them on electroencephalography (EEG) [9]. As a result, neurophysiological techniques are used to support diagnosis, but the gold standard, continuous video EEG, is not available in all UK neonatal units, and the logistics of siting leads, checking recording quality, starting the monitor, and interpreting the EEG, 24 h a day, 7 days a week are enormous. Instead, amplitude integrated EEG (aEEG) is used routinely on neonatal units, particularly in term babies with hypoxic ischaemic encephalopathy (HIE) [17, 18], and can detect 1/3 of single seizures and 2/3 of repetitive seizures, missing those that are brief or distant from the EEG leads [17, 19]. Having two channels and the single lead EEG trace available for review on the aEEG monitor improves seizure detection rates [19–21].

Once a diagnosis of neonatal seizures has been made, health care professionals have to decide what the likely aetiologies are, which investigations to perform, whether to treat the seizures and, if so, with what drugs. One particular conundrum is whether to treat electrographic seizures. There is little published data on how health care professionals view this, but 36.7% of US health care professionals presented with a theoretical case treated isolated electrical seizures in neonates with mild HIE, and 74.6% treated recurrent electrographic seizures in moderate HIE. Variation was also noted in how long neonatal seizures had to last before treatment was initiated and whether the baby was given a single dose or started on regular maintenance doses [22].

In the UK, there is no universally accepted guideline on the management of neonatal seizures, although the World Health Organisation recommends electrographic seizures should be treated in the same way as clinical seizures [23]. Little data exists on the variation in UK management of neonatal seizures, nor the reasons for any observed variation. Once treatment is commenced, Phenobarbital is recommended as first line treatment in the UK [5], although little data exists on health care professionals’ views of its effectiveness, what their choice of second line treatment is, nor how they choose when to treat and with what drug.

Our aims were the answer the following questions:

- What factors influence health care professionals when diagnosing neonatal seizures?
- How many health care professionals utilise aEEG when diagnosing neonatal seizures and what were health care professionals’ views of its use?
- How many health care professionals routinely treated clinical and electrographic seizures and what factors lead them to treat a neonate with anti-convulsant drugs?
- What anti-convulsant drugs are health care professionals using to treat neonatal epileptic seizures and in what order?
- Why health care professionals choose the drugs they do, and what are their attitudes on their effectiveness and side effects?
- What steps or evidence are needed to improve confidence in diagnosis and treatment of neoantal seizures,and to reduce variation in care between health care professionals when treating neonatal epileptic seizures?

## Methods

The mixed nature of our research questions (containing interconnected quantitative and qualitative features) required the use of a study design integrating both quantitative and qualitative methodologies [24]. We adopted an explanatory sequential mixed methods approach in two distinct phases (QUAN→QUAL) [25]. We conducted a questionnaire survey (Phase I) to examine health care professionals’ practice when diagnosing and treating neonatal epileptic seizures, followed by qualitative interviews (Phase II) to explore the reasoning for any variation noted. Equal weighting was given to both aspects of this approach.

### Phase I

We designed a questionnaire in paper and electronic versions on health care professionals’ views of neonatal seizures (Additional file 1, available online) based on the published findings of variation in care in the US and Sweden [22, 26] and the authors’ observations of UK practice. We included specific questions about levetiracetam because our experience is levetiracetam is being increasingly used in clinical practice and recommendations in the UK have suggested it could be incorporated into guidelines [5]. 196 Neonatal units were identified from a national transport group website (ukntg.net/uk-neonatal-units), which lists all UK neonatal units. The clinical lead of each unit was asked if they and other members of their staff would complete the questionnaire. Members of the British Paediatric Neurology Association (BPNA) were asked to complete the electronic version of the questionnaire via a monthly e-newsletter. Frequencies and percentages of answers were calculated and copies of seizure guidelines were requested.

Intermediate stage - connection of the two phases. The last question of the questionnaire asked if responders were willing to attend a qualitative interview to explore their views in more depth. From the list of volunteers, we used a purposeful sampling approach to ensure we obtained a range of views from different specialities, geographical areas, and years of experience. As such, the identities, specialisms and year of registration on the Specialist Register of the General Medical Council were available to us. Adhering to the explanatory sequential mixed methods design, the results of the questionnaire guided the choice of questions in the interview schedule, with a focus specifically on attitudes to the diagnosis of neonatal epileptic seizures and the timing and choice of anti-convulsant drug treatment.

### Phase II

Written informed consent was obtained from interview participants. Data was collected with semi-structured interviews conducted by a single member of the research team (LG) at a time and location of the participants’ choice. The topic guide for the interviews is available online (Additional file 2). Interviews were digitally recorded, transcribed verbatim and checked for accuracy. Thematic analysis was performed as per Braun and Clarke (2006) [27]. This included familiarisation of data, initial coding of all data using an inductive approach by two researchers (LG and ARH), review of initial codes, agreement on a coding structure for the whole dataset, and identification of a thematic structure to determine main and subthemes. Themes were developed using an iterative process to capture all range of views. We ceased recruiting for interviews when we reached thematic saturation, mindful of published recommendations on cohort size [28–30]. NVivo for Mac version 12 (QSR International PTY Ltd., 2018) was used to aid data analysis. Finally, the results of the quantitative and qualitative phases were integrated to find explanations for any observed variations in practice. This is presented in the discussion section of this manuscript.

Ethical approval was obtained from the University of Sheffield (Reference Number 017700).

## Results

### Phase I: quantitative data on neonatal seizures

One hundred questionnaires were returned: 81 consultants, 7 nursing staff, 1 trainee, and 11 unknown staff members. Thirty-three worked in neonatal intensive care (Level 3) units, 45 in local neonatal (level 2 or 1) units or paediatric departments, and 22 in paediatric neurology. The 78 responders who worked in neonatal units represented 68 different units, which is 34.7% of all UK units caring for neonates. The BPNA includes members from many specialities, including neurophysiologists, disability paediatricians and allied health care professionals, around the world, all of whom will have the option of receiving the e-newsletter. Reporting response rates for this distribution list is not appropriate as the project was not relevant to all subscribers. There are 120 consultant paediatric neurologists in the UK, so 22 responses represent 18.3% of this total population, although not all of these consultants are involved in the care of neonates with seizures. All answers were treated equally, irrespective of the degree of seniority or specialism of the respondent.

The results of our questionnaire are summarised in Table 1. 34.0% of responders routinely treated electrographic seizures, 49.0% treated them sometimes, and 17.0% reported they did not treat electrical seizures. 53.0% thought that electrical seizures were as important as clinical seizures, compared to 16.0% who thought they were less important. When asked whether seizures cause harm to the brain independent of the underlying aetiology, 62.0% thought they did and 15.0% did not. The frequency of replies for neonatologists and neurologists, as well as for only the responders identifying themselves as consultants, is shown separately in Table 1.

**Table 1:** Frequency of responses to questions in our questionnaire separated by primary type of unit in which responders work

Question		Responders				
		All responders	Working predominately in a NICU - Level 3	Working in paediatrics, Level 2 unit, or interest in epilepsy	Working predominately in paediatric neurology / neurodisability unit	All Consultant Responders
Do you treat clinical seizures (i.e. where there is no available aEEG / EEG data to confirm abnormal movements are seizures?	Yes	65/100 (65.0%)	18/33 (54.6%)	38/45 (84.4%)	9/22 (40.9%)	54/81 (66.7%)
	No	9/100 (9.0%)	4/33 (12.1%)	3/45 (6.7%)	2/22 (9.1%)	5/81 (6.1%)
	Sometimes	26/100 (26.0%)	11/33 (33.3%)	4/45 (8.9%)	11/22 (50.0%)	22/81 (27.2%)
Do you treat electrical seizures (i.e. diagnosed on aEEG/EEG) which do not have any clinical features to see?	Yes	34/100 (34.0%)	14/33 (42.4%)	15/45 (33.3%)	5/22 (22.7%)	27/81 (33.3%)
	No	17/100 (17.0%)	1/33 (3.0%)	15/45 (33.3%)	1/22 (4.5%)	11/81 (13.6%)
	Sometimes	49/100 (49.0%)	18/33 (54.6%)	15/45 (33.3%)	16/22 (72.7%)	43/81 (53.1%)
Do you think electrical seizures are:	As important as clinical seizures	53/100 (53.0%)	17/33 (51.5%)	25/45 (55.6%)	11/22 (50.0%)	43/81 (53.1%)
	More important than clinical seizures	9/100 (9.0%)	2/33 (6.1%)	4/45 (8.9%)	3/22 (13.6%)	6/81 (7.4%)
	Less important than clinical seizures	16/100 (16.0%)	6/33 (18.2%)	5/45 (11.1%)	5/22 (22.8%)	14/81 (17.3%)
	I don’t know	22/100 (22.0%)	8/33 (24.2%)	11/45 (24.4%)	3/22 (13.6%)	18/81 (22.2%)
Do you think seizures themselves cause harm to the brain/ development (i.e. not related to apnoea/hypoxia and independent of the underlying cause)?	Yes	62/100 (62.0%)	24/33 (72.7%)	27/45 (60.0%)	11/22 (50.0%)	53/81 (65.4%)
	No	15/100 (15.0%)	3/33 (9.1%)	9/45 (20.0%)	3/22 (13.6%)	12/81 (14.8%)
	I don’t know	23/100 (23.0%)	6/33 (18.2%)	9/45 (20.0%)	8/22 (36.4%)	16/81 (19.8%)
Do you routinely use cerebral function monitoring (aEEG) for monitoring neonates at high risk of seizures or those having recurrent seizures?	We use it in all neonates at risk of seizures	44/94 (46.8%)	20/31 (64.5%)	12/43 (28.0%)	12/20 (60.0%)	39/78 (50.0%)
	We use it only in those with HIE	10/94 (10.6%)	5/31 (16.1%)	5/43 (11.6%)	0/20 (0.0%)	9/78 (11.5%)
	We use it in selected cases, HIE and non-HIE	20/94 (21.3%)	5/31 (16.1%)	8/43 (18.6%)	7/20 (35.0%)	14/78 (18.0%)
	We don’t use it at all	19/94 (20.2%)	1/31 (3.3%)	17/43 (39.5%)	1/20 (5.0%)	16/78 (20.5%)
	I don’t know	1/94 (1.1%)	0/31 (0%)	1/43 (2.3%)	0/20 (0%)	0/78 (0%)
With reference to Phenobarbital, do you think it is …	Very effective	29/94 (30.9%)	6/31 (19.4%)	16/43 (37.2%)	7/20 (35.0%)	25/78 (32.1%)
	Stops some seizures but not all	65/94 (69.1%)	25/31 (80.6%)	27/43 (62.8%)	13/20 (65.0%)	53/78 (67.9%)
	Not at all effective	0/94 (0%)	0/31 (0%)	0/43 (0%)	0/20 (0%)	0/78 (0%)
Have you tried Levetiracetam for treatment of neonatal seizures?	Yes	65/94 (69.1%)	26/31 (83.9%)	19/43 (44.2%)	20/20 (100%)	59/78 (75.6%)
	No	29/94 (30.9%)	5/31 (16.1%)	24/43 (55.8%)	0/20 (0%)	19/78 (24.4%)
Do you think Levetiracetam (Keppra) is …	Very effective	21/65 (32.3%)	8/26 (30.8%)	9/19 (47.4%)	4/20 (20.0%)	19/59 (32.2%)
	Stops some seizures but not all	44/65 (67.7%)	18/26 (69.2%)	10/19 (52.6%)	16/20 (80.0%)	40/59 (67.8%)
	Not at all effective	0/65 (0%)	0/26 (0%)	0/19 (0%)	0/20 (0%)	0/59 (0%)
Compared to Phenobarbital, do you think Levetiracetam is	Better than phenobarbital	14/65 (21.5%)	7/26 (26.9%)	3/19 (15.8%)	4/20 (20.0%)	13/59 (22.0%)
	As good as phenobarbital	17/65 (26.2%)	3/26 (11.5%)	9/19 (47.4%)	5/20 (25.0%)	14/59 (23.7%)
	Less good than phenobarbital	6/65 (9.2%)	1/26 (3.9%)	1/19 (5.2%)	4/20 (20.0%)	6/59 (10.2%)
	I don’t know	28/65 (43.1%)	15/26 (57.7%)	6/19 (31.6%)	7/20 (35.0%)	26/59 (44.1%)

73/94 (77.7%) responders’ units had a guideline for the management of neonatal seizures. 90 (95.7%) responders indicated their guideline’s first line anti-convulsant medication: 83/90 (92.2%) used phenobarbital, 1 (1.1%) phenytoin, 2 (2.2%) used either phenobarbital or phenytoin, 1 (1.1%) levetiracetam, and 3 (3.4%) either phenobarbital or levetiracetam. In addition to phenobarbital, a range of other drugs were used to treat neonatal seizures (Table 2).

**Table 2:** Other anti-convulsant drugs responders reported they used

Drug	Proportion of responders saying they had experience of using in neonates (*n* = 94)
Phenytoin	74 (78.2%)
Levetiracetam	65 (73.3%)
Midazolam	62 (66.0%)
Lorazepam	18 (19.2%)
Paraldehyde	13 (13.8%)
Lignocaine	15 (16.0%)
Vitamins / pyridoxine	12 (13.3%)
Diazepam	5 (5.3%)
Clonazepam	4 (4.4%)
Topiramate	3 (3.3%)
Carbamazepine	3 (3.3%)
Sodium valproate	1 (1.1%)
Vigabatrin	1 (1.1%)
Prednisolone	1 (1.1%)

29/94 (30.1%) responders reported that phenobarbital was very effective at treating neonatal seizures, 65 (69.2%) said that it stopped some seizures but not all. No responder thought that phenobarbital was ineffective.

65 (73.3%) responders had experience of using levetiracetam, and their views on its effectiveness were similar to phenobarbital (Table 1). When asked to directly compare levetiracetam to phenobarbital, 14/65 (21.5%) thought levetiracetam was more effective, 17 (26.2%) equally effective, 6 (9.2%) less effective, and 28 (43.1%) did not know. Reported side effects seen with levetiracetam were:

- None 39/49 (79.6%)
- Irritability, hyperkinetic movements / jitteriness 5 (10.2%)
- Sleepiness 3 (6.1%)
- Electrolyte disturbance 2 (4.1%)
- Respiratory depression 1 (2.0%)

**Fig. 1:**
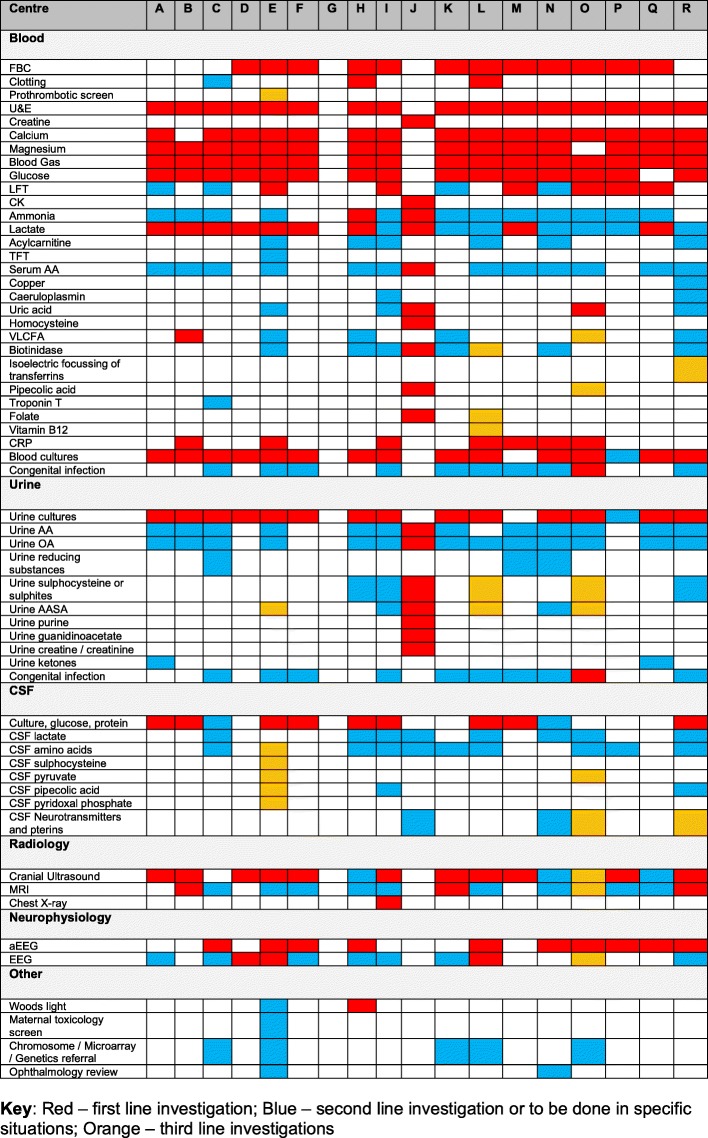
Investigations recommended on received guidelines to determine the aetiology of neonatal seizures: Red – 1st line; blue – 2nd line or only to be requested under certain circumstances; orange – 3rd line. Guideline from centre G did not attempt to recommend investigations. Abbreviations: FBC – full blood count; U&E – urea and electrolytes; LFT – liver function tests; CK – creatine kinase, TFT – thyroid function test; AA – amino acids; VLCFA – very long chain fatty acids; CRP – C-reactive protein; OA – organic acids; AASA – alpha amino adipic semialdehyde; MRI – magnetic resonance imaging; aEEG – amplitude integrated electroencephalography; EEG – electroencephalography

**Fig. 2:**
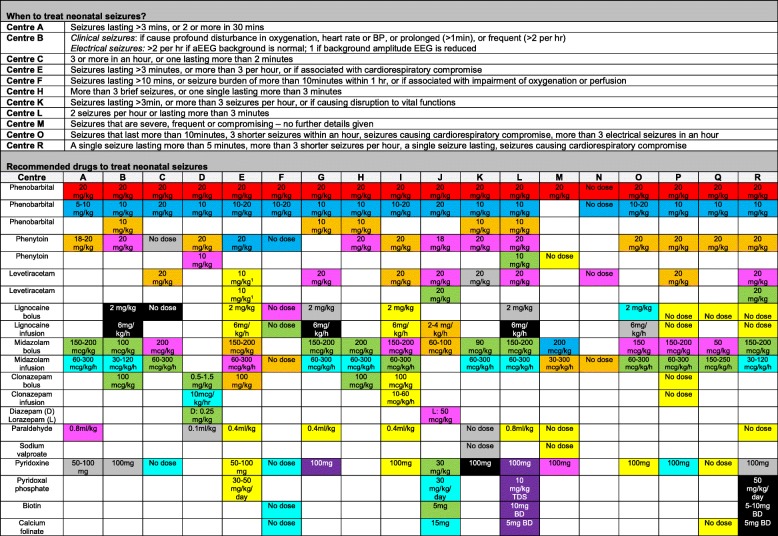
Information from received guidelines on when to treat neonatal seizures, and recommended treatments and doses. Red – 1st line; blue - 2nd line; orange – 3rd line; pink – 4th line; green – 5th line; turquoise – 6th line; grey – 7th line; black – 8th line; purple – 9th line; yellow – to be tried at the discretion of the consultant at any time. Abbreviations: D – diazepam; L – lorazepam; mg – milligram; mcg – microgram; kg – kilogram; h – hour; d- day, BD – twice a day; TDS – three times a day

We received 18 different neonatal unit or network guidelines. Network guidelines included a number of centres of different levels, some of which will and will not have on-site access to aEEG and EEG. Variation was noted in recommended aetiological investigations for neonatal seizures (Fig. 1), guidance on when to treat seizures, and choice of anti-convulsant drugs if phenobarbital was ineffective (Fig. 2). Thirty-eight responders agreed to consider being involved in Phase 2.

### Phase II: qualitative data on the diagnosis and treatment of neonatal seizures

Sixteen consultants were interviewed: 5 Level 3 Neonatologists, 7 Paediatric Neurologists, 4 Paediatricians at a local neonatal (level 2) unit / district general hospital: 3 with expertise in neonatology and 1 in epilepsy. Eleven were male and 5 female. We chose interviewees from different geographic regions to ensure we were not finding views linked only to local practice. The mean years of consultant experience was 11 years and 2 months (range 2 months to 28 years). The length of interviews ranged from 45 min to 1 h 40 min, with a median length of 1 h 10 min. Pseudonyms are used to maintain anonymity.

**Fig. 3:**
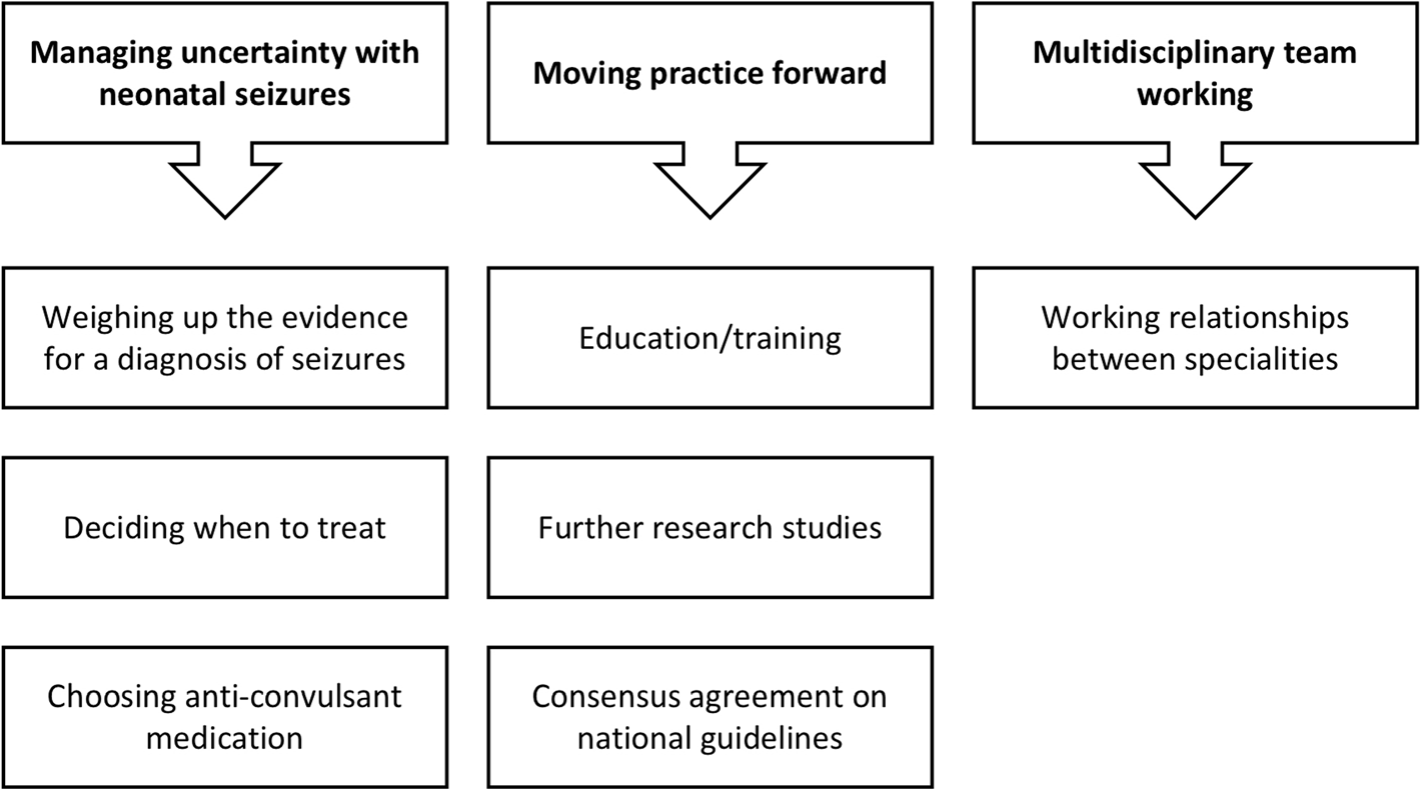
Summary of results of thematic analysis from qualitative interview study

Three themes emerged from the study (Fig. 3):

- Managing uncertainty with neonatal seizures
- Moving practice forward
- Multidisciplinary team working.

#### Managing uncertainty associated with neonatal seizures

This theme explored the uncertainty clinicians face when deciding whether a neonate is having seizures and, if so, how to treat them. It contained the following subthemes:

- weighing up the evidence for a diagnosis of seizures
- deciding when to treat
- choosing anti-convulsant medication.

##### Weighing up the evidence for a diagnosis of seizures

Consultants found diagnosing neonatal seizures difficult, and consultants reported uncertainty increases with experience and seniority because of a greater awareness of differential diagnoses. Consultants were suspicious of diagnoses made by junior staff, particularly doctors in training, who were seen as over-diagnosing seizures and being too influenced by nursing staff. Lucy, a neonatologist, explained:

*“You’re much more persuaded by the nursing staff as a junior doctor cos you totally trust them … . So, if they said to you ‘that baby had a seizure’ you probably would have been much more likely to go along”.*

When a consultant was sceptical about a diagnosis, they wanted descriptions of the events to confirm the diagnosis of a seizure was correct. Tim noted a cultural difference in how junior doctors obtain descriptions of neonatal events to older children and adolescents:

*“If you are sitting in an outpatient clinic and the GP [General Practitioner] has made a referral that this patient has had episodes of funny movements and they are worried they might be seizures, the thing that you put great emphasis on is the history. You listen to what the parent has to say, they may have taken a video of it on their phone.... Flip to a post-natal ward, nobody believes a parent - ever. So, if a parent presents their baby having funny movements, a midwife will not believe anything until she has seen it for herself. Bizarrely, the doctors, who if they were in paediatrics would have no problem going on the maternal story, will not believe the maternal story until they have seen it for themselves. And you have to ask yourself ‘what is it that so changes their attitude to being in paediatrics and being in neonates?’”*

The consultant paediatric neurologists particularly lamented the quality of the descriptions they received and noted nearly all seizures were described as *“tonic clonic”.* Bella, a Consultant Paediatric Neurologist, noted:

“*We will be told that they’re fitting and when you ask for a description it is often hard to get specific detail about that. So, it sometimes is hard to know whether what they are describing is a true seizure or whether it is an involuntary movement or some other, um, neurological phenomenon.”*

Christopher noted that junior colleagues gave him the seizure type rather than a description:

*“I think it’s difficult with medical colleagues, isn’t it? If they say ‘it’s clonic’, I probably wouldn’t quiz them in great detail about whether their limbs are stiff and consistent, you know what I mean? … I would probably be a bit more inclined to accept at face value their interpretation of the seizure type, but of course that might incorrect.”*

One way to improve diagnosis suggested by both neonatologists and neurologists was to video events on smartphones, although one interviewee was worried doing this on the neonatal unit might imply care was suboptimal: *“It’s obviously an attractive idea I just feel it makes you look a little bit sort of silly if you’re in intensive care unit and say ‘oh can you video as well’, but you know perhaps I shouldn’t be, perhaps it’s my own pride”.*

EEG or aEEG were also used to improve diagnostic certainty. Neurologists preferred to use a combination of history and EEG with video but were concerned about the quality of neonatal EEG recordings and reporting, and noted some neurophysiologists lacked confidence and competence to interpret neonatal EEG. On the other hand, neonatologists struggled with the accessibility of EEG, as Fiona explained:

*“The unit has got semi-direct access to an EEG machine and even that sometimes takes us a day. … And then in other units, I have only just learned this, because we were discussing the network seizure guideline, that they didn’t have access to EEG machines at all. I was a bit shocked actually.”*

Instead, neonatologists saw aEEG as a pragmatic solution for improving diagnostic certainty, but acknowledged it was *“not the answer to your prayers”* because it missed brief seizures and those distant from the leads. Consultants highlighted a number of training needs including: how to site the leads, set up the monitor, and interpret the trace. Neonatologists correctly perceived neurologists were wary of aEEG: neurologists thought its introduction had *“jumped the gun”* before the optimal way to manage neonatal seizures was understood. A proportion of the neurologists’ scepticism reflected their own lack of confidence in interpreting aEEG, as Suresh explained:

*“I’m not particularly competent or confident from my perspective. I don’t, we never used it in my training.”*

Both neurologists and neonatologists suggested time-locked video alongside aEEG would improve diagnosis, but such monitors were not readily available.

##### Deciding when to treat neonatal seizures

Once a seizure was diagnosed, consultants weighed-up the risks and benefits of medication. The aetiology of seizures was critical: seizures related to a severe neonatal epilepsy syndrome, structural brain abnormalities, or metabolic conditions were not treated as aggressively. Where the seizures were caused by HIE, the following factors were important when deciding whether to treat:

- The natural history of the seizures, i.e. acute symptomatic seizures “burn out”
- Whether the seizures were accompanied by significant clinical features like profound apnoea / desaturation
- Frequency and duration
- Whether electrical seizures were as important as clinical seizures
- Whether seizures cause harm independent of the underlying aetiology
- The side effects of the anti-convulsant drugs
- Parental or nursing staff anxiety.

There were clear benefits of treating seizures with severe clinical features. For clinical seizures with milder manifestations or electrical seizures, most consultants made a judgement on a *“case-by-case basis”* about whether the frequency and duration were sufficient to warrant treatment, agreeing to treat *“a high burden of seizure activity”*. Interviewees explained there was no agreement about what a high burden of seizure activitiy meant and scientific evidence was not a part of clinician thinking, with variation seen even within single units, as Fiona explained:

“*I have not got any set rules. Within the unit we really have not got any set rules. Um, we’ve all got slightly different thresholds from when we would treat, really depending on the clinical scenario.”*

A number of neonatologists held onto algorithms they were taught as trainees, as Tim explained:

*“We used to have the Levene rule of 3. So, you needed to have 3 seizures or a seizure lasting more than 3 minutes, before we would treat, okay, I think that was in an hour”.*

A proportion of neonatologists aggressively treated both electrical seizures and clinical seizures with minor manifestations, driven by a belief that all seizures directly contributed the “*burden of brain injury”.* Neurologists were more comfortable not treating electrical seizures, as Bella explained:

*“The question is, ‘Is it better to have a more normal background EEG if you can, but a child that’s really flat and moribund because you’ve got them on 5, 6 drugs?’. … in certainly my training, it was very much ‘treat the child or the infant, not the EEG’”.*

Suresh summed up the consultant neurologists’ thoughts by saying:

*“the link between seizure frequency and severity and brain development is weak and difficult. We don’t really understand it, why do some children do very badly with their development and others do well.”*

Consultants of all specialities also considered the risk of anti-convulsants affecting neuro-developmental outcome themselves. Most thought adverse neuro-developmental outcome was more likely to occur after long-term administration of anti-convulsants, rather than isolated doses, and were sceptical about relying on animal study data*.* Some consultants were concerned the sedative properties of traditional drugs made a neonate’s *“neurological examination probably seem more abnormal than it may be”*, making *“their progress or lack of it”* impossible to determine and inpatient stays longer. Ben noted:

*“The problem with the phenobarbitone yes, so the half life time was 5-7 days. So, we treat HIE babies with phenobarb, maybe for electrical seizures, and then you have to tell the parents ‘Well, because we had to treat with this, it will take the baby at least 3 days to wake up and to come back to normal and that is not because the brain is damaged’.”*

If initial drugs are ineffective, consultants appraised the balance between the benefits of treatments and side effects again, but many consultants avoided multiple drugs because of sedative side effects and the need for ventilatory support. If several drugs did not work, consultants reached a *“plateau”* where they accepted seizures rather than using further drugs.

The final driver that consultants perceived pushed them towards deciding to treat neonatal seizures was parental or staff anxiety. They wanted to *“feel like we’re doing something”,* although this could lead to them *“treating the parents and the nurses rather than the baby”.* As Lucy explained:

*“I think there’s sometimes pressure, I mean that in a nice way, not in you know not a bad way at all, from nursing staff to get to get rid of all funny movements.”*

##### Choosing anti-convulsant medication

Phenobarbital was the first line drug for all of our interviewees because of tradition, familiarity, and local or network guidelines*,* as Ben explained:

“*Neonatology people are quite traditional in what they use … you will find that most people stick to what they know, what they use, and therefore they will always follow the guidelines.”*

Neonatologists thought it was *“inappropriate”* to deviate unilaterally from the guideline and thought they would face questions or criticisms from nursing staff or colleagues if they did. This reduced their experience of alternative treatments, because seizures that had not responded to initial anti-convulsant drugs were more likely to be refractory to other medications too.

Neurologists reported they were not often involved in initiating treatments for neonatal seizures. When they were involved in treatment decisions, neurologists were less protocol-driven, would tailor drug choice to underlying aetiology, and were more comfortable with a wider repertoire of drugs than neonatologists. Neurologists liked levetiracetam, having had experience of its use in older children, and thought it was effective at treating neonatal seizures. John was an exception, noting *“We use quite a lot and I, the more I have used it, I think it might not be working as good as I expected it to work. … Maybe the dose is not right”.* Few side effects were noted with levetiracetam in neonates, and it was described by neurologists as *“clean”* and *“forgiving”.* Overall, neurologists couldn’t “*see a reason why it can’t be first line.”*

#### Moving practice forward

This theme examined how practice could be improved in the future. There were three main suggestions: education; further research studies; and consensus agreement on national guidelines.

Training for medical trainees, consultants and nurses on the neurological assessment of the neonate, patterns of movements likely to be seizures, differential diagnoses, how to set up and interpret aEEG, the use of EEG, and aetiological investigations was reported to be *“the most important thing”* to be doing now. In comparison, research was seen as more important for long-term improvements. Consultants formed two groups: ‘purists’ insisted on evidence from randomised controlled trials on drug effectiveness, side effects, how aggressively to treat seizures, whether to treat electrical seizures, and the long-term developmental effects before they would change guidelines. The second group were ‘Pragmatists’, who accepted organising formal studies was problematic, expensive, and would take a long time. They suggested drawing on the cumulative experience of clinicians to form a national consensus guideline. Ben explained:

*“It’s not research, it’s just identifying ‘what is happening?’ and ‘What are people doing and why are they doing it?’ This is exactly what you want to understand, and you need to understand why people feel safe or what is needed to make them feel safe … it’s not about research knowing why levetiracetam might be better, but if you can just point out the new doctrine has same efficacy but they wake up earlier, so the parents are more pleased about whatever this is about it, and then you can set up a new strategy including all these aspects … I think that this is ending up may be in a national survey and a guideline and that might be influential … Neonatologists are not brave. It’s not like neurosurgeons: you give them a new toy and they will stick the toy in the head of a patient.”*

#### Multidisciplinary team working

This theme described working relationship between specialities. Neonatologists worked in networks with other centres, but only a small number worked regularly with neurologists. When they did, they gained *“more insight maybe into seizures, and how, what we should treat”*.

Neurologists were not routinely called when seizures were first treated and were only consulted in a child who was not responding as the neonatologists expected. Neurologists thought they could be consulted more frequently and saw positives in collaboration: they felt deskilled when they were only consulted for complex cases, leading to *“book based”* advice, and wanted to see a wider spectrum of conditions. They thought their experience on a wide range of neurological conditions, drugs, and developmental outcome would be useful for neonatologists and reported cross fertilisation of *“information from conferences”* and experience would promote creativity. One neurologist suggested the neonatologists have wide experience of a limited number of conditions and, without formal neurology training, have a blinkered view: “*I think sometimes people don’t know what they don’t know”*.

## Discussion

Consultants face many challenges when considering the cause of abnormal neonatal movements and need to reach a “point of certainty” before making a diagnosis of a seizure. A number of factors increase this degree of certainty, the main one being the ability to witness the events themselves. This is a challenge because consultants are typically called to review a baby after the event has stopped and find that junior medical or nursing staff give bland “seizure types”, which are often wrong, instead of detailed descriptions of what happened. This may be a result of a cultural difference between neonatology and general paediatrics / neurology, where ictal phenomenology and the use of videos is an essential part of the diagnostic process. As a result, consultants find an abnormal neonatal event has often been attributed and treated as a seizure before they attend, without consideration of differential diagnoses. This, and the traditional classifications of seizures types in neonates, as described by Volpe [31], may explain to some extent why over-diagnosis of neonatal seizures is common and why consultants think diagnostic accuracy could be improved without neurophysiology support using a thorough approach to diagnosis. Even if it this is true, it would not improve the diagnosis rates of the large majority of neonatal seizures, which have either subtle, brief or no clinical features at all [9], supporting calls for neonatal seizures to be reliant on neurophysiological techniques [15, 32, 33].

Where neurophysiological techniques are used in neonates, consultants disagree on which method is the most suitable. Nearly all tertiary neonatology services routinely use aEEG, compared to 39.5% from secondary level neonatal and paediatric units. Although we did not plan our interview schedule to discuss the use of aEEG in secondary level units, one paediatrician in a district general hospital was actively seeking to purchase a monitor, whilst another thought it was inappropriate for their unit to have one because their staff lacked training and expertise in its use. Neonatal and neurology networks need to decide whether they support the introduction of aEEG into secondary level units and general paediatric wards. From a practical perspective, it is feasible with appropriate training and support [34], but others argue the National Institute of Clinical Excellence recommend young children with seizures should be reviewed by a paediatric neurologist [35], and aEEG may delay access to specialist opinion. Away from secondary level centres, neurologists prefer EEG and are wary of aEEG, thinking neonatologists place undue reliance on its ability to detect neonatal seizures. Some of this negativity may reflect neurologists’ own lack of training and confidence in using aEEG. We should also be aware that, whilst EEG is seen as the gold standard investigation for the diagnosis of neonatal seizures, it is only as good as the neurophysiologist interpreting it, and differences in opinions on what EEG findings are neonatal seizures will exist in practice [36, 37]. In contrast, neonatologists recognise the value of EEG but do not have the same ease of access as neurologists, taking a pragmatic view that aEEG is a flawed tool, but one that allows for monitoring over longer periods of time than EEG, is more likely to capture recurrent seizures, is accessible, relatively easy to interpret, and better than clinical diagnosis on its own.

A further area of controversy relates to whether electrographic seizures are important or not. Tertiary neonatologists are twice as likely as neurology consultants to treat all electrographic seizures. This observation cannot occur because neurologists think electrographic seizures are unimportant; in fact, twice as many neurologists as neonatologists reported in our questionnaire that electrical seizures were more important than seizures with clinical features. Instead, it probably reflects whether consultants think seizures cause additional harm to the brain independent of the underlying aetiology: almost three quarters of neonatal staff answering our questionnaire thought seizures cause harm to the developing brain, compared to half of neurology staff, and neonatal interviewees indicated this was a major driver for them to treat electrical seizures aggressively.

The published evidence on whether clinical or electrical seizures causes harm in neonates is unclear: animal studies are contradictory about whether induced seizures are associated with brain injury without hypoxia-ischaemia [38, 39], and one study shows seizures and hypoxia-ischaemia in rats combine to produce worse brain injury [39]. In neonates with HIE, near infrared spectroscopy demonstrates increased cerebral oxygenation, blood flow and oxygen metabolism during seizures [40, 41], and MR spectroscopy results are affected by seizure severity [42]. Evidence on whether seizures are associated with poor outcome is similarly contradictory. Small studies show that treating clinical and electrical seizures using aEEG and / or EEG is associated with improved MRI scores at discharge compared to treating only clinically suspected seizures [43, 44]. Whilst there no statistically significant difference in developmental assessment is noted between groups at 18-24 months of age, one study shows a trend to better outcomes when neurophysiological techniques were used and electrographic seizures treated [44]. Another study found the presence of clinical seizures without neurophysiological confirmation is associated with worse outcomes at 4 years of age when the severity of MRI abnormalities is controlled for [45]. Larger studies, however, have shown the association between the presence of seizures and outcome is complex [46–48]. One group found there is no clear link between the presence of seizures and outcome in HIE, but there is an association between increasing seizure frequency and duration and outcome [46]. A large retrospective cohort study using a national insurance database found that neonatal seizures are associated with greater risk of epilepsy and intellectual disability later in life, independent of the aetiology of the neonatal seizures [48]. None of these studies show conclusively that aggressive treatment of neonatal electrical seizures improves outcome.

Another explanation why neonatologists are more likely than neurologists to treat electrical seizures aggressively relates to the aetiologies they see: neonatologists commonly see acute symptomatic seizures, so anti-convulsant drug use is short-lived and within the realm of neuroprotection. Neurologists see more refractory neonatal seizure, epilepsy, and a wider range of seizure types and aetiologies, so treatment is more likely to be unsuccessful, of longer duration, and with greater risk of side effects. The published literature on whether short term anti-convulsant drug use is harmful to neonates is controversial: anti-convulsant drugs are associated with neuronal apoptosis and inhibited neurogenesis in animal models [49, 50] but little is known about this relationship in humans. Levetiracetam and topiramate are less implicated [51, 52], and topiramate may be neuroprotective [53, 54]. A single retrospective study found increasing doses of phenobarbital for neonatal seizures is associated with worse cognitive outcome and cerebral palsy than levetiracetam [55], and long-term treatment with phenobarbital in children with febrile convulsions is associated with cognitive difficulties [56, 57].

When neonatal seizures do have clinical features, there is variation in when consultants commence anti-convulsant treatments: neonatologists who think seizures cause harm treat them aggressively; others wait until the baby has had “enough” seizures to warrant treatment. The timeframe for this decision is entirely arbitrary and relates to dogmatic rules consultants were taught as trainees. When a decision is made to treat neonatal seizures, the choice of first line drug is almost always phenobarbital because of familiarity and tradition. Published evidence shows that phenobarbital stops between 28 and 63% of neonatal seizures [11, 58–61], so all neonatologists have seen it work and feel comfortable with its side effects. The downside of this approach is that it stops neonatologists gaining experience of other drugs and, on the rare occasions they do use alternatives, it is as second or third-line treatment in naturally more refractory seizures. Neonatologists feel they cannot unilaterally change their first-line drug choice because they worry about what their colleagues would think if they broke with ingrained, guideline-driven practice. Some neonatologists look towards neurologists for their experience of alternative drugs and want a national discussion to share experiences and reach a consensus on drug treatment.

Neurologists have greater experience of a wide range of drugs, and choose their first line treatment based on seizure type and aetiology; for example, epileptic spasms are treated with steroids and / or vigabatrin and tonic seizures related to benign familial neonatal seizures with carbamazepine. For acute symptomatic seizures, neurologists also choose phenobarbital first because of tradition, but are more comfortable than neonatologists using newer drugs, like levetiracetam. We found genuine equipoise in both groups on whether Levetiracetam or Phenobarbital is more effective, although neurologists are more likely to report Levetiracetam as less effective, perhaps reflecting the aetiologies they see. Reassuringly, few reported side effects are seen with Levetiracetam, as nearly 80% of questionnaire responders reporting Levetiracetam had either no or mild side effects, reflecting similar data from small studies [55, 60–66]. Interviewees report they would like to change their first line treatment to Levetiracetam if it was found to be equally as effective as phenobarbital with less side effects, because phenobarbital’s sedative properties may prolong hospital stays.

We found extreme variation in the choice and dosage of second-line, third-line and subsequent drugs for neonatal seizures. Our interviews reveal that consultants do not know which drugs are the most effective and rely on the local traditions and network guidelines that often reflect the personal preference of their local expert. Both specialities report the need larger-scale studies into seizure treatments, but acknowledge their methodology and logistics are problematic.

Finally, neurologists in our study reported they want closer collaboration with neonatologists to share knowledge and experience. Neonatologists are more focussed on working in neonatal networks, and only the interviewees who had close liaison with neurology colleagues saw the value of closer relationships. Currently, services appear disparate. Promoting training and collaboration between the two specialisms could improve care for neonates, drive forward developments in education, and help standardise care across the UK. An alternative model could be the formation of Neonatal Neurology Intensive Care Units [67], but it remains to be seen if this is the optimal method of delivering care in the UK given large numbers of neonates are at risk of neurological complications.

There are limitations to our data. Our response rate for the questionnaire is reasonable, but there is no way to determine whether the views and practices of individuals who responded are the same as those who did not. Some responders worked in the same units as others, and it is possible that the culture of specific units where multiple questionnaires were returned influenced the interpretation of our questionnaire results. We only interviewed consultants because the decision to investigate and treat ultimately resides with them, but the views of junior medical and nursing staff are important as they are “first-line” when recognising abnormal movements and seizures. We would have need a larger sample size to reach data saturation as the range of views would have been larger, and we did not have the resources to do this. It is a potential future area of research to determine if the views of consultants are substantially different from other members of staff. We also acknowledge that the responders to our questionnaire in Phase one were from a mixture of staff members, so the data from Phase One may not be directly transferable to the results of our interviews. However, 81.0% of questionnaire responders were consultants, so the effect of having other health care professionals answer the questionnaire is likely to be small. We purposefully chose consultants from different units, specialities, sex and experience levels to obtain as wide a range of their views as possible, but we cannot comment on the views of consultants who did not volunteer to be interviewed. Therefore, as with any qualitative interview study, we cannot guarantee our results are generalisable to all consultants managing neonatal seizures. Finally, we are aware that medicine, where possible, should be evidence-based. There are many limitations to relying on clinicians’ perceptions of what they think they do, which can be very different from their practice in real life, and the effectiveness of drug therapies. However, understanding perceptions is important because they explain why some people follow (or not) evidence and guidelines, and why clinicians make the choices they do when evidence is limited or of poor quality, as with the treatments of neonatal seizures. Where good quality exists, it is important it is followed because perceptions may be wrong.

## Conclusion

Health care professionals face many uncertainties when diagnosing, investigating and treating neonatal seizures, resulting in wide variations in practice throughout the UK. Our data is the first to reveal the views of paediatricians and the challenges they face, along with the solutions they suggest. These include: development of nationwide educational packages to improve the descriptions taken of neonatal seizures and aEEG interpretation; increased use of video; improved access to neurophysiology investigations; a national discussion on whether aEEG should be available in level 2 units or if evaluation of all neonates with suspected seizures should be centralised to level 3 units; and closer collaboration between neurology and neonatal teams to drive forward a national consensus guideline, which would standardise the management of neonatal seizures across the UK.

## Supplementary information

**Additional file 1.** questionnaire sent to neonatal and paediatric neurology centres.

**Additional file 2.** topic guide used for qualitative interviews.

## Data Availability

The data from the quantitative questionnaire and qualitative interview phases are not publicly available to maintain confidentiality of centres and individuals, as per ethical approval. However, all reasonable requests for information will be provided on request to the corresponding author.

## Abbreviations

aEEG: Amplitude integrated electroencephalography
BPNA: British Paediatric Neurology Association
EEG: Electroencephalography
GP: General Practitioner
HIE: Hypoxic ischaemic encephalopathy
QUAL: Qualitative data or methods
QUAN: Quantitative data or methods
