# Supplementary material for: Investigating and managing neonatal seizures in the UK: an explanatory sequential mixed methods approach
Source: BMC Pediatr. 2020 Jan 28;20:36. doi: 10.1186/s12887-020-1918-4 (PMC6986085; doi:10.1186/s12887-020-1918-4)
Supplement: Supplementary file 1 — Additional file 1. questionnaire sent to neonatal and paediatric neurology centres. [file 12887_2020_1918_MOESM1_ESM.pdf]

Survey on Treatments of Neonatal Seizures in the UK

Thank-you for completing this questionnaire on the treatment of neonatal seizures. Ethical approval has been gained from the University of Sheffield. This work forms part of a MSc degree for Lucy Gosling, Medical Student.

All answers will be treated confidentiality, and we will not publish your unit's name in any reports written.

Name: ☐

What type of unit do you work at? ☐

Which centre do you work in? ☐

1. Do you treat clinical seizures, i.e. where there is no available CFM / aEEG / EEG data to confirm abnormal movements are seizures?

Yes ☐ No ☐ Sometimes ☐

2. Do you treat electrical seizures (i.e. diagnosed on aEEG / CFM / EEG) which do not have any clinical features to see?

Yes ☐ No ☐ Sometimes ☐

3. Do you think electrical seizures are:

As important as clinical seizures ☐ More important than clinical seizures ☐ Less important than clinical seizures ☐ I don't know ☐

4. Do you think seizures themselves cause harm to the brain / development (i.e. not related to apnoea / hypoxia and independent of the underlying cause)?

Yes (Go to Q5) ☐ No (Go to Q6) ☐ I don't know (Go to Q6) ☐

5. If yes, please explain why (please use back of paper if necessary):

6. Do you routinely use cerebral function monitoring (aEEG) for monitoring neonates at high risk of seizures or those having recurrent seizures?

Yes, we use it in all neonates at risk of seizures ☐ We use it only in those with HIE ☐ No, we don't use it at all ☐ We use it in selected cases only, HIE and non-HIE ☐ I don't know ☐

7. What is your first line anti-epileptic drug (and dose):

8. What other drugs do you use routinely to treat neonatal seizures?

Phenobarbital	☐	Phenytoin	☐
Levetiracetam (Keppra)	☐	Diazepam	☐
Lorazepam	☐	Midazolam	☐
Lignocaine	☐	Paraldehyde	☐

Others (please state):

9. With reference to phenobarbital, do you think it is…

Very effective at treating seizures ☐ Not at all effective at treating seizures ☐ Stops some seizures, but not all ☐

10. Have you tried Levetiracetam for treatment of neonatal seizures? Yes (Go to Q11) ☐ No (Go to Q13) ☐

11. Do you levetiracetam (Keppra) is…

Very effective at treating seizures ☐ Not at all effective at treating seizures ☐ Stops some seizures. but not all ☐

12. Compared to phenobarbital, do you think levetiracetam is:

Better than phenobarbital ☐ As good as phenobarbital ☐ Less good than phenobarbital ☐ I don't know ☐

13. What side effects are you worried about / have you seen with levetiracetam in neonates?

14. Do you have a seizure treatment guideline? Yes ☐ No ☐

15. If yes, will you send us a copy of your guideline? Yes ☐ No ☐

16. Would you be willing to agree to an interview about your thoughts on treating neonatal seizures in more detail?

Yes ☐ No ☐

If yes to Q15 or Q16, please provide contact phone number or email:
