# Supplementary material for: Investigating and managing neonatal seizures in the UK: an explanatory sequential mixed methods approach
Source: BMC Pediatr. 2020 Jan 28;20:36. doi: 10.1186/s12887-020-1918-4 (PMC6986085; doi:10.1186/s12887-020-1918-4)
Supplement: Supplementary file 2 — Additional file 2. topic guide used for qualitative interviews. [file 12887_2020_1918_MOESM2_ESM.docx]

**General opening question**

Can you tell me more about your job and what situations you might see a neonate with seizures?

**Recognising neonatal seizures**

In your experience, how easy do ***YOU*** find it to recognise seizures in neonate?

- Is that the same for junior doctors and nurses or do they find it easier or harder?
- What challenges do you or the neonatal staff face in recognising neonatal seizures?
- What could help make it easier to recognise seizures in a neonate?
- What are the sort of things you see when a neonate is having a seizure?

What experience do you have of amplitude EEG (also called cerebral function monitors – CFM) in neonates?

- Where or when did you learn to interpret CFM?
- How confident do you find interpreting CFM? Why do you say that?
- Can you tell me what sort of things you look for when you’re interpreting CFM?
- What are the challenges you find with interpreting CFM?
- How well CFM detects seizures in your experience? Why do you say that?

**When to treat neonatal seizures**

When you think back of cases you have seen, what makes you decide to treat neonatal seizures?

- Are there any seizures you wouldn’t treat? Why?
- Are some seizures OK to just sit on and not treat? How many does a baby need before you treat them? Where does that plan come from?
- I’m interested in what people called subclinical or electrical seizures, and I mean those that are detected on EEG or CFM but have no clinical features. Do you treat those?
  - When do you decide to treat them?
  - Why do you think it is (OR is not) important to treat them?

**Electrical subclinical seizures and outcome**

- Where a neonate has seizures after a brain injury, like HIE, meningitis or trauma, do you think treating electrical / subclinical seizures makes a difference?
  - Some people suggest the seizures themselves cause further brain injury. What do you think?
  - (IF yes) I’m interested in what the mechanism might be for how further injury happens…what do you think?
  - How did you come to that opinion?
  - Can you tell me about any disadvantages you have found in treating electrical / subclinical seizures?

**Anti-convulsant drugs**

- Can you tell me about what drugs you use to treat neonatal seizures?
  - What are your first and second line drugs? Why?
- How well do you think the standard drugs, like phenobarbital or phenytoin, work?
- Do you think any particular drug is better than the others? If so, why?
- What are your thoughts about the side effects of anti-convulsants in neonates?
  - What about them makes you concerned?
- Have you used levetiracetam (or Keppra) in neonates? Tell me about your experience of using it?
  - Did you use it first line? Second? Third?
  - How well did it work?
  - Do you think it’s as good, better or worse than phenobarbital or phenytoin? Why?
  - What side effects that you noted with it?

**Closing questions**

- It seems that there is a lot of uncertainty about the best way to treat neonatal seizures. What is needed to help improve that uncertainty?
  - If research, what kind of studies would be useful?
- If you were talking to a junior doctor and had one piece of important information you wanted them to take away about neonatal seizures, what would it be?
- Is there something else that you think we should have discussed about neonatal seizures but haven’t?
